# Supplementary material for: The Effect of the Gallbladder Environment during Chronic Infection on Salmonella Persister Cell Formation
Source: Microorganisms. 2022 Nov 16;10(11):2276. doi: 10.3390/microorganisms10112276 (PMC9698170; doi:10.3390/microorganisms10112276)
Supplement: Supplementary file 1 [file microorganisms-10-02276-s001.zip › Supplementary Table S1.pdf]

Supplementary Table S1. Strains used in this study.

**Laboratory strains**					**MIC (µg/mL)**	
**Strain**	**Characteristics**			**Source**	**Cipro**	**Kan**
JSG4383	*S*. Typhi Ty2; *rpos*+			Gift of Roy Curtis	0.5	24
JSG210	*S*. Typhimurium 14028			ATCC	0.125	24
**Clinical Isolates**						
**Strain**	**Isolation source/characteristics**	**Infection type**	**Country of origin**	**Source**		
JSG3074	Gallstone	Chronic	Mexico	General Hospital of Mexico, Mexico City	2	32
JSG3076	Gallstone	Chronic	Mexico	General Hospital of Mexico, Mexico City	2	16
JSG3400	Bile	Acute	USA	Ohio Department of Health	2	64
JSG3441	Stool	Acute	USA	Ohio Department of Health	2	64
JSG3979	Gallbladder	Chronic	Vietnam	Gift of S. Baker	2	32
JSG3981	Gallbladder	Chronic	Vietnam	Gift of S. Baker	2	16
